# Management of Acute Myeloid Leukemia: A Review for General Practitioners in Oncology

**DOI:** 10.3390/curroncol29090491

**Published:** 2022-08-30

**Authors:** Ryan J. Stubbins, Annabel Francis, Florian Kuchenbauer, David Sanford

**Affiliations:** 1 Leukemia/BMT Program of British Columbia, Vancouver Coastal Health, BC Cancer, Vancouver, BC V5Z 1M9, Canada; 2 Department of Medicine, Division of Hematology, University of British Columbia, Vancouver, BC V5Z 1M9, Canada; 3 Leukemia/BMT Program of British Columbia, Vancouver Coastal Health, Fraser Health, Vancouver, BC V5Z 1M9, Canada; 4 Terry Fox Laboratory, British Columbia Cancer Research Centre, Vancouver, BC V5Z 1L3, Canada

**Keywords:** acute myeloid leukemia, targeted therapy, chemotherapy, stem cell transplant, palliative care

## Abstract

Acute myeloid leukemia (AML) is a hematologic malignancy that most frequently develops in older adults. Overall, AML is associated with a high mortality although advancements in genetic risk stratification and new treatments are leading to improvements in outcomes for some subgroups. In this review, we discuss an individualized approach to intensive therapy with a focus on the role of recently approved novel therapies as well as the selection of post-remission therapies for patients in first remission. We discuss the management of patients with relapsed and refractory AML, including the role of targeted treatment and allogeneic stem cell transplant. Next, we review non-intensive treatment for older and unfit AML patients including the use of azacitidine and venetoclax. Finally, we discuss the integration of palliative care in the management of patients with AML.

## 1. Introduction

Acute Myeloid Leukemia (AML) is an aggressive malignancy of the white blood cells that leads to symptoms related to bone marrow failure and organ infiltration. Untreated, AML is a universally fatal condition and life-threatening complications can quickly develop in asymptomatic patients. Our group has recently reported an incidence of AML in British Columbia of 4.11 per 100,000 population per year [1], which is similar to what has been reported in recent SEER data (4.34 per 100,000) [2]. AML is relatively rare, making up ~1.1% of cancer diagnoses [2], however it is the second most common form of leukemia and accounts for ~1.9% of cancer deaths [2]. AML is a cancer that predominantly affects middle aged and older adults and >2/3 of cases occur in patients over age 50 years, with a median age at diagnosis of 68 years [2].

Significantly, AML is associated with a relatively poor survival and recent Canadian Cancer Society statistics (2019) report a 5 year overall survival (OS) of 21%, which is similar to highly lethal solid organ malignancies, such as lung, liver, and brain cancer (19%) [3]. Nevertheless, outcomes in AML are strongly linked to the genetic characteristics of the disease and the treatment received and prognosis varies significantly between patients. In this review, we will discuss the current diagnosis of AML, prognosis, and current management of adult AML patients with intensive and non-intensive approaches. We do not cover the management of acute promyelocytic leukemia (APL) or pediatric AML, which are beyond the scope of this review.

## 2. Diagnosis and Prognosis

### 2.1. How Can AML Present, How Is AML Diagnosed and What Are Important Initial Tests?

The presentation of AML can vary from an asymptomatic patient with an abnormal complete blood count (CBC) to a patient with critical illness requiring immediate ICU support. Accordingly, making a diagnosis of AML should be a medical urgency even in a stable patient. Most patients present with symptoms related to bone marrow failure (e.g., fever or infection, symptoms related to anemia, or bruising or bleeding symptoms) and an abnormal CBC showing cytopenias, leukocytosis and/or circulating blasts. Organ infiltration can also occur at diagnosis with gums, with lymph nodes and skin being frequent sites; rarely, AML can present as an isolated solid tumor (myeloid sarcoma). Central nervous system involvement (CNS) at diagnosis is less common compared to acute lymphoblastic leukemia (ALL) and retrospective studies report incidences ranging from 1.7–5% [4]. However, the presence of neurologic symptoms requires prompt investigation with CNS imaging and lumbar puncture to evaluate for this.

Diagnosis of acute leukemia requires a bone marrow sample showing ≥20% blasts, typically assessed on a bone marrow aspirate by morphology [5]. The diagnosis of AML is made by flow cytometry or immunohistochemistry to demonstrate an immunophenotype consistent with an immature blast cell population and cell surface or cytoplasmic markers of myeloid lineage. Notably, AML can be diagnosed with <20% blasts if the recurrent genetic abnormalities (except t (9;22) (q34.1;q11.2)/BCR::ABL1) are identified [6]. At the time of the diagnostic bone marrow for suspected AML, samples should be collected for cytogenetic and molecular testing as this information is essential to guide disease classification, prognostication, and treatment selection.

Specific medical emergencies that can occur in the early stages of diagnosis and treatment include tumor lysis syndrome (TLS), hyperleukocytosis with leukostasis, and complications of cytopenias (bleeding, infections, anemia). TLS occurs when the rapidly dividing myeloid blasts break down, either spontaneously or in response to therapy, resulting in a large release of phosphate, potassium, and uric acid into the serum. It is important to monitor AML patients for this complication (lactate dehydrogenase (LDH), electrolytes, phosphate, uric acid, renal function) early in their disease course. Most AML patients should receive prophylactic allopurinol and intravenous fluids to prevent renal complications from TLS, and patients with active TLS should be considered for rasburicase, which breaks down uric acid. Leukostasis from hyperleukocytosis usually occurs in patients with high peripheral white blood cell counts (usually >50 × 10^9^/L), although this can be seen at lower levels; patients with leukostasis usually present with symptoms resembling ischemia (e.g., stroke-like symptoms, visual symptoms, or chest pain), hypoxia, or confusion. If the leukostasis symptoms are severe, the patient may require emergency leukapheresis (mechanical removal of leukocytes from the blood), though frequently patients can be managed with leukoreduction (e.g., hydroxyurea) alone. Complications related to cytopenias are more common; all AML patients should receive appropriate blood product support and regular CBCs/coagulation studies to ensure adequate platelet, hemoglobin, and fibrinogen levels, and broad-spectrum intravenous antibiotics should be initiated if fevers or infectious symptoms are detected [7].

### 2.2. Assessing Prognosis in AML

Prognosis in AML is dependent on several factors, although one of the strongest is the underlying genetics of a patient’s AML. An adaptation of the widely used European Leukemia Network (ELN 2022) prognostic system is shown in Table 1, which divides AML into favorable, intermediate, and adverse risk categories [6]. This classification is based on cytogenetic changes detected on karyotype or FISH as well as single gene mutations. In this system, mutations in certain genes are associated with a better prognosis (e.g., *NPM1*, some *CEPBA* mutations) and others are associated with a less favorable prognosis (e.g., *RUNX1*, *ASXL1*, *TP53*). In addition, the ELN categorization incorporates specific gene–gene interactions to estimate prognosis (e.g., patients with *FLT3-ITD* have a relatively better prognosis with a concurrent *NPM1* mutation) [6].

While prognostic scoring systems, such as the ELN 2022, are useful to guide risk-adapted therapy for patients with AML, they do not adequately predict outcomes in older patients and those receiving non-intensive treatment [8]. Several other patient-related factors are important to consider when estimating prognosis. Subgroups, such as therapy-related AML (t-AML) or AML arising from a prior myeloid neoplasm (i.e., secondary AML), have a worse prognosis than de novo AML. In addition, outcome of patients in this group is still impacted by disease genetics [9]. Response to initial treatment is another important prognostic factor and patients that do not enter remission after 1–2 cycles of intensive chemotherapy have a poor outcome [10]. Even in patients entering morphologic remission, the presence of measurable residual disease (MRD) has been consistently associated with a worse outcome and is an independent prognostic factor for relapse and OS in many studies [11].

## 3. Current Management of AML in the First-Line

### 3.1. Frontline Intensive Treatment in Younger Fit Patients

**Figure 1:**
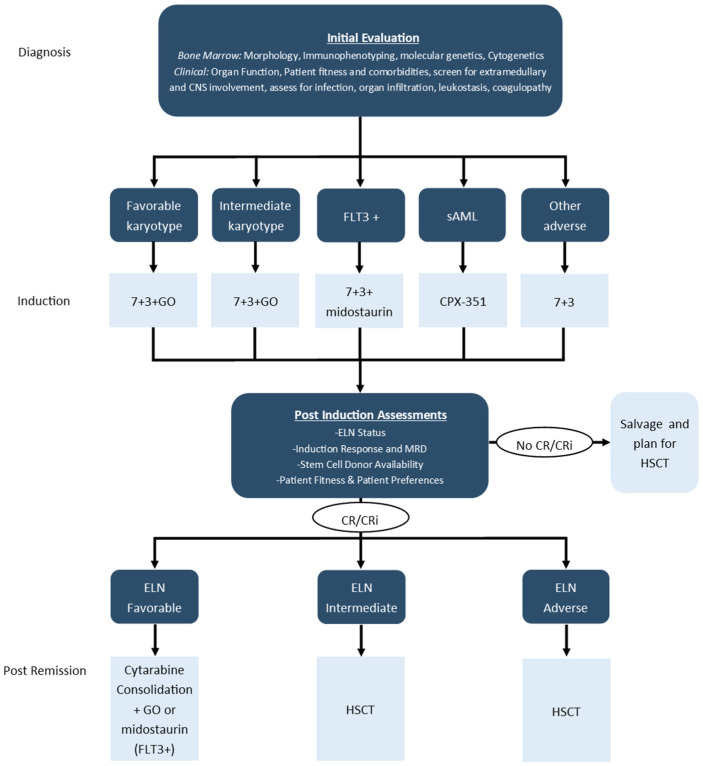
Overview of frontline intensive treatment of AML.

Until relatively recently, genetics or AML sub-type did not significantly impact front-line induction chemotherapy. Historically, most centers would treat all eligible and fit AML patients with a standard combination of an anthracycline (usually daunorubicin or idarubicin) IV bolus given for 3 days and continuous infusion cytarabine given for 7 consecutive days, which came to be known as “7 + 3” chemotherapy. Over about the last 5 years, this has changed with the approval of several new agents in the front-line treatment setting, including midostaurin for patients with *FLT3*-mutations, gemtuzumab-ozogamicin for patients with intermediate or favorable risk karyotype and CPX-351 for patients with secondary AML. An approach to frontline treatment incorporating these agents is shown in Figure 1 and a summary of the pivotal phase 3 randomized controlled trials (RCTs) supporting these approvals is shown in Table 2 [12,13,14].

Midostaurin is an oral FLT3 and multikinase inhibitor that is approved for newly diagnosed AML with *FLT3-ITD* or *TKD* mutation and is given following intensive induction and consolidation chemotherapy on days 8–15 of a treatment cycle. A large, phase 3 RCT showed improved OS and event-free survival (EFS) with midostaurin vs. placebo in this setting. Midostaurin is generally well tolerated, with the most common side effect being a drug-related rash; the QTc interval must also be monitored while patients are on midostaurin [12].

Gemtuzumab-ozogamicin (GO) is an antibody-drug conjugate targeting CD33, which is a surface marker expressed on the majority of AML blast cells. The drug has been studied in several large prospective RCTs using different doses and dosing schedules; not all of these studies showed an OS benefit and some reported higher rates of toxicity compared to the control arm [19]. A large individual patient level meta-analysis helped to clarify the role for this agent and showed a large OS benefit (5 year OS: 77.5% vs. 55%) in patients with favorable risk cytogenetics (t (8;21) or inv (16)/t (16;16)), a more modest OS benefit in patients with intermediate risk cytogenetics (5 year OS: 40.7% vs. 35.5%) and no difference in OS in those with adverse risk cytogenetics [20]. Notably, this meta-analysis reported less toxicity with a lower dose of 3 mg/m^2^ with equivalent efficacy outcomes when compared to the higher 6 mg/m^2^ dose utilized in earlier trials [20].

CPX-351 is a liposomal formulation of 7 + 3 chemotherapy with a synergistic 5:1 molar ratio of cytarabine to daunorubicin that improves outcomes in patients with secondary AML compared to standard induction and consolidation chemotherapy [21]. In a phase 3 RCT, which enrolled older patients (age 60–75) with secondary AML, CPX-351 improved response rates as well as OS compared to standard “7 + 3” chemotherapy [13,16]. The 5-year OS was still low in both arms (18% vs. 8%), highlighting the adverse-risk prognosis of secondary AML. Interestingly, patients’ who received CPX-351 and underwent HSCT had improved OS relative to patients who received 7 + 3 that also underwent HSCT (56% vs. 23%).

This shift in the frontline, intensive treatment of AML patients has resulted in a requirement for a more rapid turn-around of cytogenetic and *FLT3* testing (e.g., ~<1 week) to facilitate appropriate therapy selection. For some patients, it may be necessary to have a short delay while deciding on the appropriate treatment, and large retrospective studies suggest this approach does not adversely impact outcomes in selected stable patients [22,23]. In the coming years, it is possible other targeted agents will be included with frontline therapy given the recent promise of drugs such as IDH1/2 inhibitors, menin-inhibitors, BCL2-inhibitors, and new monoclonal antibodies [24]. Therefore, we anticipate that there will be an evolving requirement for rapid turn-around of genetic testing including next-generation gene sequencing panels to allow for timely classification of AML subtype and identification of treatment targets.

### 3.2. Post-Remission Treatment in Younger Fit Patients

The initial goal of induction treatment is to obtain a complete remission (CR), which is defined as <5% blasts on a cellular bone marrow aspirate, count recovery (e.g., absolute neutrophil count (ANC) > 1 × 10^9^/L, platelets > 100 × 10^9^/L), with no circulating blasts or evidence of extramedullary disease. Achievement of blasts <5% in the bone marrow without recovery of ANC > 1.0 and platelets >100 is termed CR with incomplete count recovery (CRi) and has been associated with worse outcome compared to CR in some reports [25,26]. Remission is an important treatment target as it is generally a requirement for delivery of curative-intent post-remission therapy such as cytarabine-based consolidation chemotherapy or hematopoietic stem cell transplant (HSCT). HSCT is the most potent post-remission treatment to reduce relapse, however the OS benefit with this strategy is partly offset by increased non-relapse related mortality (NRM), which is usually estimated ~20% in relatively fit groups of patients [27]. In general, HSCT is considered in first CR (CR1) in patients with intermediate or adverse risk AML but not in those with favorable risk disease [28]. This approach has partially developed from the results of older prospective donor vs. no-donor trials, suggesting OS benefit in these subgroups and is reflected in most major working group guidelines [6,29].

Several other important factors other than disease characteristics are also necessary to consider when recommending allogeneic HSCT to an individual patient, including donor availability, patient fitness and comorbidities, conditioning regimen, and patient preferences. Comorbidity can be assessed using the hematopoietic comorbidity index (HCT-CI) and higher scores using this tool are associated with increasing risk of non-relapse mortality (NRM) [30]. Patient fitness and frailty may be formally assessed through comprehensive geriatric assessment, which is a multi-faceted evaluation that includes standardized measurements of physical performance and cognitive testing. When feasible, use of a myeloablative conditioning regimens is preferred over reduced intensity or non-myeloablative conditioning as this approach reduces relapse and improves OS [31].

Patients who are older or those with non-favorable risk AML undergoing intensive chemotherapy who are not eligible for HSCT are expected to have high rates of relapse. Oral azacitidine (CC-486) maintenance has been recently approved for patients, who achieve remission following intensive chemotherapy and are ineligible for HSCT [17]. In the phase 3 RCT investigating CC-486 maintenance AML patients with intermediate or adverse risk cytogenetics age 55 years or older, following intensive induction or consolidation chemotherapy, were randomized within 4 months of CR/CRi to CC-486 or placebo. The study found significantly improved OS (24.7 months vs. 14.8 months) as well as RFS (10.2 months vs. 4.8 months). The treatment was associated with higher rates of neutropenia, infection, and GI side-effects, although health related QoL did not appear to be impaired with CC-486.

### 3.3. Non-Intensive Approaches in Older and Unfit Patients

As the majority of patients diagnosed with AML are older, a significant proportion are unable to receive intensive chemotherapy due to poor tolerance of these treatments. Historically, outcomes for these patients have been quite poor [1]. Until recently the main treatment options for this group of patients have been non-intensive chemotherapies, such as low-dose cytarabine (LDAC) and azacitidine along with supportive care including transfusion support, pain control, antibiotics and palliative care referral. Azacitidine has been shown previously to modestly improve OS in older AML patients compared to conventional care treatments [32,33].

More recently, the combination of azacitidine and the BCL2 inhibitor venetoclax has been approved by Health Canada [34,35]. Azacitidine and venetoclax have been reported to be superior to azacitidine and placebo in a large multi-centre RCT [18]. The primary endpoint of the study was OS, which was longer in the azacitidine and venetoclax arm vs. the control group (14.7 vs. 9.6 months, HR, 0.66; 95% CI, 0.52 to 0.85; *p* < 0.001). Response was also improved with azacitidine and venetoclax vs. azacitidine and placebo (CR/CRi 66.4% vs. 28.3%, *p* < 0.001) and the median time response was shorter (1.3 vs. 2.8 months). The combination of azacitidine and venetoclax was associated with greater myelosuppression along with a higher rate of febrile neutropenia compared with the control arm. The occurrence of TLS, a known complication with venetoclax in other disease indications, was relatively uncommon with only 3 patients (1%) experiencing laboratory tumor lysis in the azacitidine and venetoclax arm. The combination of LDAC and venetoclax has also been compared to LDAC and placebo in a phase 3 RCT [36]. This study found a higher rate of CR/CRi with LDAC and venetoclax (48% vs. 13%); however, there was no statistically significant difference in OS (Median OS 7.2 months vs. 4.1 months, 0.75 (95% CI, 0.52–1.07; *p* = 0.11).

Due to this data, the combination of azacitidine and venetoclax has become a new standard of care for older patients and/or those ineligible for intensive chemotherapy. Practice points related to use of this regimen and supportive care are shown in Table 3. Despite a significant improvement in survival with the introduction of azacitidine and venetoclax, this treatment alone is not considered curative and all patients will eventually progress after an initial response. For this reason, ongoing enrollment of older patients onto clinical trials is essential to improving outcomes for this group of patients and there are several promising novel agents and combinations now being studied [24]. With the introduction of azacitidine and venetoclax, the precise role of single agent azacitidine, decitabine and LDAC is unclear. It is possible that for some patients, these agents may be more tolerable than azacitidine and venetoclax and LDAC can be delivered at home, which is an important factor for some patients.

## 4. Management of Relapsed and Refractory AML

### 4.1. Approach to Relapsed/Refractory Disease

Despite the previously outlined advances in upfront therapy for AML, approximately 20% of younger patients treated with intensive chemotherapy approaches will have primary refractory disease, defined as ≥5% blasts on end of treatment bone marrow; this can be upward of 40% in older (≥60 years) patients [38,39]. Additionally, depending on underlying disease biology/genetics and the consolidation approach utilized, approximately 30–50% of patients who achieve CR1 will relapse after consolidation with chemotherapy alone [40], and approximately 20–30% of patients who receive allogeneic HSCT will relapse [41]. Relapsed/refractory (R/R) disease is usually detected on either a planned follow-up marrow examination or after declining blood counts are observed on monitoring CBCs. Generally, all patients with suspected R/R disease should undergo a repeat bone marrow examination to confirm the presence of R/R disease, rule-out alternate diagnoses, and collect samples for repeat cytogenetic and/or molecular testing to document clonal evolution and potentially targetable genetic changes [6]. Upon confirmation of the presence of R/R disease, a full repeat clinical evaluation should be performed, specifically including an assessment of performance status, laboratory studies (e.g., electrolytes, uric acid, lactate dehydrogenase), appropriate organ function assessment (e.g., LVEF assessment), and HLA-typing, if not performed at diagnosis.

The outcomes for patients with R/R AML are broadly poor, though several clinical (e.g., age, performance status) and disease-related (e.g., cytogenetics) factors make each patients’ situation unique. Patients who have primary refractory disease after induction regimens with high-dose chemotherapy have a poor prognosis, with only approximately 20–30% entering a second CR after repeat (salvage) high-dose cytarabine-based chemotherapy and, historically, approximately 10% being long-term survivors [38]. Outcomes amongst patients with relapsed AML who previously achieved a first CR with high-dose chemotherapy alone are more heterogeneous; patients with a longer relapse-free interval (>6 months), ELN-favorable risk cytogenetics, and a younger age at relapse (≤45 years), who did not receive upfront HSCT can have second CR rates upward of 50%, with repeat high-dose chemotherapy [42]. In contrast, patients with none of these features have outcomes more comparable to primary refractory disease, with long-term survival rates of <20% [42]. Patients who relapse after allogeneic HSCT also have a particularly poor outcome, with only a small fraction achieving another remission with additional therapy and a median survival of between 3–4 months, without additional therapy [43].

As such, new approaches that can improve outcomes for R/R AML patients are needed, and this is a very active area of research. The selection of a treatment approach for R/R AML patients must be individualized, though many of these patients will proceed to a second high-dose chemotherapy regimen, if sufficiently fit and judged likely to benefit. The exact re-induction regimen utilized is highly institution dependent; some commonly used chemotherapy protocols include MEC (Mitoxantrone, etoposide, cytarabine), FLAG-IDA (Fludarabine, cytarabine, idarubicin, G-CSF), and other high-dose cytarabine based regimens (e.g., high-dose cytarabine with etoposide), all of which demonstrate comparable results with second CR rates of between 20–40% across all patient groups [44]. It is also important to be aware that patients with R/R AML are at higher risks of treatment-related complications, and should be closely monitored for cardiac complications and prophylactically receive a mold-active antifungal agent (e.g., Posaconazole) [45].

### 4.2. The Role for Repeat FLT3 Testing and Gilteritinib

It is important to recognize that, in patients with R/R AML, the cytogenetic and mutational profiles can change from the time of diagnosis and are not necessarily fixed. In particular, the gain or loss of mutations in the driver gene *FLT3* that were either not present or detectable at diagnosis has been well described [46,47]. This is particularly relevant as targeted agents are now available for AML patients with *FLT3* mutations and it has been reported that loss of FLT3-ITD mutations occur in ~40% of patients with R/R AML after treatment with midostaurin [47]. As such, R/R AML patients should have a repeat molecular assessment for the presence of a *FLT3* mutation, either through single-gene testing or a repeat next-generation sequencing panel.

One targeted agent that has been approved for R/R AML patients is gilteritinib, an oral agent that is both a potent and selective inhibitor of FLT3 [15,48]. The approval of gilteritinib came from the ADMIRAL study, a multicenter, phase-3, randomized, placebo-controlled clinical trial of gilteritinib versus salvage chemotherapy for R/R AML patients with mutations in either *FLT3-ITD*, *FLT3-TKD* D835, or *FLT3-TKD* I836 [15]. The majority of these patients had received anthracycline-containing induction regimens prior to enrollment (83.8%), and only a minority had previously received a *FLT3* inhibitor (13.2%) as part of their induction regimen (e.g., midostaurin) [15]. Amongst 371 enrolled patients, the overall response rate (CR + CRi) was 34.0% for the gilteritinib arm and 15.3% for the salvage chemotherapy arm. This translated into an improved median OS of 9.3 months in the gilteritinib arm versus 5.6 months for salvage chemotherapy (HR 0.64, 95% CI 0.49–0.83, *p* < 0.001) [15]. Patients receiving gilteritinib should have monitoring of their liver enzymes and QT interval; peripheral edema, skin rash, and differentiation syndrome are some specific toxicities associated with this drug. Gilteritinib also appears to have activity in patients who previously received midostaurin, with response rates of 58% and a survival of 7.8 months reported in retrospective cohorts [15].

### 4.3. The Role for Inhibitors of Isocitrate Dehydrogenase (IDH) 1 and 2

Mutations in either of the isocitrate dehydrogenase (*IDH*) genes, *IDH1* or *IDH2*, are found in 10–15% of patients at the time of AML diagnosis, and can rarely be acquired at the time of relapse [46]. These mutations alter the metabolism of the AML cells, driving clonal expansion and chemotherapy resistance. Oral, targeted inhibitors have been developed for both the IDH1 (ivosidenib) and IDH2 (enasidenib) enzymes. Ivosidenib has shown activity as a single agent in a phase 1 dose-escalation and expansion trial, producing an overall response rate of 41.6% and median duration of response 8.2 months (95% CI 5.5–12.0 months) in R/R AML patients [49]. The combination of ivosidenib with azacitidine was tested in the phase 3, randomized, placebo-controlled AGILE trial. Amongst 146 patients with R/R AML, the combination of ivosidenib with azacitidine produced a CR rate of 47% (vs. 15% for azacitidine with placebo) with a median overall survival of 24 months (vs. 7.9 months for azacitidine with placebo) [50].

Enasidenib has also been studied as a as a single-agent in R/R IDH2-mutated AML; it was tested in a phase 3, randomized IDHentify trial, producing a higher overall response rate (41% ensidenib versus 11% conventional care regimen), but with a disappointing OS result (6.8 months enasidenib versus 6.2 months conventional care regimen) [51]. Enasidenib seems to have more efficacy in combination with azacitidine; in an open-label, phase 1b/2 trial, 101 patients with R/R AML were randomized to enasidenib with azacitidine versus azacitidine alone. The overall response rate was 74% in the enasidenib with azacitidine group versus 36% in the azacitidine alone group [52]. Further studies will be needed to fully define the role for enasidenib in R/R AML. The IDH1/2 inhibitors are unique in that they act primarily by inducing cellular differentiation, rather than being directly cytotoxic [53]. One unique toxicity that results from this is differentiation syndrome, which can result in volume overload, coagulopathy, and leukocytosis. Another notable toxicity of the IDH inhibitors is abnormal liver enzymes and hyperbilirubinemia, necessitating regular monitoring of liver function [49].

### 4.4. The Role for Hypomethylating Agents with Venetoclax

Hypomethylating agents (azacitidine and decitabine) and venetoclax have also been studied in patients with R/R AML, which is currently off-label use. There is interest in this combination, given the relatively high remission rate and relatively tolerable safety profile in older adults, in the frontline setting. The use of a hypomethylating agent in combination with venetoclax in the R/R AML setting has been shown to produce response rates of between 12–51% in retrospective series, though significant heterogeneity is seen based on clinical (e.g., previous hypomethylating agent exposure) and genomic (e.g., higher response rate with *IDH1/2* and *NPM1* mutations) features [54,55,56,57,58]. Reported OS is quite short in most of these retrospective series (usually ranging between 3–6 months) and further prospective clinical trials are required to better define the role of this treatment in R/R AML.

### 4.5. The Role for HSCT

Allogeneic HSCT plays an important role for eligible and fit patients with R/R AML, and it generally represents the only chance for achieving a sustained remission in this patient population. It is important to begin the process of planning for allogeneic HSCT early in the patient’s disease course as often the window to proceed to this therapy is limited, particularly in the R/R setting. At most centers, the patient will be expected to have achieved a second CR before proceeding to allogeneic HSCT, as outcomes for patients proceeding to HSCT with active disease are poor [59]. The majority of R/R AML patients who are judged fit to proceed to allogeneic HSCT and have an appropriate donor should receive this therapy, preferably with fully myeloablative conditioning chemotherapy if feasible, as non-transplant therapies do not have curative potential in R/R AML at present. In certain patient subsets within R/R AML, high-dose chemotherapy followed by autologous HSCT in second CR may produce long-term remissions, though autologous HSCT is not widely utilized for R/R AML in the North American context [60].

## 5. Palliative Care

### 5.1. Palliative Needs and Integrated Palliative Approaches in AML

The proliferation of novel targeted therapies and clinical trials for AML has added hope for patients and treating clinicians for improved OS [61]; yet, innovative therapies create unique challenges in balancing prolonged OS with preservation of quality of life for patients at risk of treatment-related morbidity and mortality. Even though poor OS and a high physical and psychological symptom burden continue to be major issues for AML, patients with AML are far less likely to receive integrated palliative care compared to other malignancy groups [62,63,64,65]. Common physical symptoms experienced throughout leukemia include fatigue, dyspnea, pain, nausea, and anorexia [66]. In addition, patients and families face distressing illness uncertainty, depression, anxiety, and unrealistic illness expectations [64,67,68]. Alongside the supportive care armamentarium, an integrated palliative approach service can be instrumental in reducing symptom burden, enhancing illness coping and supporting quality communication around prognostic uncertainties [62,67,68,69].

Integrated palliative approaches alongside active oncologic treatment are a considered a standard of practice in cancer care regardless of prognosis [64,70]. The prognostic uncertainties during the AML trajectory should not preclude early palliative care integration; rather, palliative approaches should be integrated based on patient needs, because upstream integration enables patients to derive the many benefits of palliative care [67,68,71]. Palliative care involvement has been shown to produce improved documentation of goals of care and timely referrals to hospice [67]. Recent qualitative studies and randomized trials revealed that integrated palliative care alongside curative intent treatment can improve the patient and family experience, significantly reduce physical and psychological symptom burden [62,67], reduce depression scores and risks of post-traumatic stress disorder [71].

### 5.2. Interdisciplinary Collaboration to Support Palliative Needs

Interdisciplinary collaboration is a foundational pillar of supportive and palliative care in AML; physical and occupational therapists support activities of daily living, dieticians assist with nutritional concerns and social workers provide instrumental emotional and practical support throughout the illness journey. Given the unpredictable nature of AML, it is imperative that physicians, nurses, and nurse practitioners initiate serious illness and goals of care conversations at clinically stable points during the illness trajectory [66,72]. Hematologist-oncologists and palliative clinicians can form a symbiotic relationship to support these medically and ethically complex conversations [67]. Palliative clinicians must be attuned to the particular features and issues encountered in AML care, such as prognostic uncertainty, goals for longer survival, unique complications, and rapid decline to death [68].

### 5.3. Barriers to Palliative Integration and Community-Based End-of-Life Care

Unrealistic expectations of a cure or long-term remission can detract from the importance of serious illness conversations [72,73]. Patients with AML develop long-term therapeutic relationships with hematology-oncology teams [72], so it becomes difficult for other specialties, such as inpatient or community-based palliative care teams to form similar rapport. Early integrated palliative care allows palliative clinicians to build longitudinal relationships with patients (similar to the relationship with treating oncology teams), such that if transition to end-of-life suddenly approaches, therapeutic rapport is already established [71]. Oncology teams often use the terms palliative care interchangeably with hospice and end-of-life care, which leads to challenges in earlier integration of palliative approaches.

Transfusion dependency typically precludes patients from receiving hospice care [71], although exceptions can be approved on a case-by-case basis. Hospice typically does not provide blood work, which can discourage patients from hospice admission at end-of-life. Patients with AML are more likely to die in hospital compared to advanced solid tumor groups [72], and this is in part due to uncontrolled infections and bleeding being common causes of mortality. However, a smaller proportion of patients may choose to receive end-of-life care at home or in hospice settings and it is essential that community care teams maintain regular communication with inpatient oncology teams to ensure continuity of care for this unique group of patients.

## 6. Summary

The landscape of treatment in AML has changed significantly over approximately a 5–10-year time period, with the approval of several new treatments. This has also increased the complexity of treatment of patients with newly diagnosed and R/R AML with an increasing emphasis on genetic testing and targeted therapies at both time points. Despite improvements in therapy, the mortality of AML remains high and patients and health care providers can benefit from improved integration of palliative care during treatment. Given the increasing numbers of older adults with AML, with aging populations, this patient group will likely represent a growing portion of community oncology practices. It is important for general practitioners in oncology and community oncologists to be aware of the spectrum of treatment options available to patients with AML. Most of these patients will benefit from a close collaborative relationship between providers at the tertiary AML treatment center within their region and community oncologists. In particular, collaboration around referrals for clinical trials, administration of some aspects of therapy and supportive care closer to home. There is ongoing innovative research in AML, and we anticipate new therapies along with personalized approaches will continue to improve outcomes for patients.

**Key Points:**

- Patients presenting with suspected acute leukemia should undergo a thorough assessment for associated complications along with an expedited diagnosis;
- Accurate diagnosis and risk stratification require cytogenetic and molecular genetic testing, and this information may guide initial treatment along with selection of post-remission therapy and use of HSCT;
- Repeat testing for FLT3-mutation is required for patients with R/R AML to guide appropriate therapy;
- Azacitidine and venetoclax is a new, more effective treatment for older patients with AML, although it is associated with increased myelosuppression requiring close monitoring and appropriate supportive care;
- Integration of palliative care during treatment can improve outcomes, symptom management, and facilitate discussions around goals of care and end-of-life planning.

## Figures and Tables

**Table 1:** ELN 2022 Risk Stratification [6].

Risk Category	Genetic Abnormality
Favorable	t (8;21) (q22;q22.1); *RUNX1-RUNX1T1* inv (16) (p13.1q22) or t (16;16) (p13.1;q22); *CBFB-MYH11* Mutated *NPM1* without *FLT3*-ITD bZIP in-frame mutated *CEBPA*
Intermediate	Mutated *NPM1* with *FLT3*-ITD Wild-type *NPM1* with *FLT3*-ITD t (9;11) (p21.3;q23.3); *MLLT3-KMT2A* Cytogenetic abnormalities not classified as favorable or adverse
Adverse	t (6;9) (p23;q34.1); *DEK-NUP214* t (v;11q23.3); *KMT2A rearranged* t (9;22) (q34.1;q11.2); *BCR-ABL1* inv(3) (q21.3q26.2) or t (3;3) (q21.3;q26.2); *GATA2*, *MECOM(EVI1)* t (3q26.2;v); *MECOM (EVI1)*-rearranged −5 or del (5q); −7; −17/abn (17p) Complex karyotype, monosomal karyotype Mutated *ASXL1*, *BCOR*, *EZH2*, *RUNX1*, *SF3B1*, *SRSF2*, *STAG2*, *U2AF1*, or *ZRSR2* Mutated *TP53*

Reprinted with permission from Ref. [6]. 2022, American Society of Hematology.

**Table 2:** Overview of Newer Drug Approvals in AML [12,13,14,15,16,17,18].

Treatment	Indication	Median OS Exp. vs. Ctrl	Selected Toxicities	Approval Status ^a^	Ref.
Midostaurin	FLT3+ Frontline with intensive chemotherapy	74.7 vs. 25.6 months	GI (nausea, vomiting, diarrhea), infection, skin rash, pulmonary toxicities, QT prolongation	HC/FDA Approved (Frontline)	[12]
Gilteritinib	FLT3 + R/R	9.3 months vs. 5.6 months	GI (nausea, vomiting, diarrhea), infection, transaminitis, increased CK, myelosuppression, QT Prolongation, differentiation syndrome	HC/FDA Approved (R/R)	[15]
Gemtuzumab-ozogamicin	Favorable/Intermediate/Unknown cytogenetics Frontline with intensive chemotherapy	27.5 vs. 21.8 months (NS)	Infection, myelosuppression and delayed platelet recovery, hepatic toxicity and VOD, infusion reactions	HC/FDA Approved (Frontline)	[14]
CPX-351	Secondary AML Frontline	9.56 vs. 5.95 months	Infection, myelosuppression, bleeding	HC/FDA Approved (Frontline)	[13]
Oral Azacitidine (CC-486)	Maintenance following intensive chemotherapy, HSCT ineligible	24.7 vs. 14.8 months	GI (nausea, vomiting, diarrhea), infection, myelosuppression	HC/FDA Approved (Post-induction maintenance)	[17]
Venetoclax	Elderly/Unfit Frontline with azacitidine	14.7 vs. 9.6 months	Infection, myelosuppression, tumor lysis syndrome	HC/FDA Approved (Frontline, induction ineligible)	[18]

^a^—HC, Health Canada; FDA, Federal Drug Administration.

**Table 3:** Practice points for azacitidine and venetoclax therapy [37].

	Considerations
Tumor Lysis Prophylaxis	- Start anti-hyperuricemic agent prior to starting therapy - Ensure adequate hydration (oral or IV) - Reduce WBC < 25 × 10^9^/L with hydroxyurea before starting - Close monitoring of electrolytes, creatinine, uric acid, calcium, phosphate during ramp-up, such as q6-8 h after each new dose level of venetoclax and 24 h after final dose
Antimicrobial Prophylaxis	- Antibacterial and antiviral prophylaxis for neutropenia (<0.5–1.0 × 10^9^/L) and consider antifungal prophylaxis - Concurrent azole antifungals (e.g., voriconazole, posaconazole, fluconazole) require dose reductions of venetoclax
Cytopenias	- Anticipate need for RBC and/or platelet transfusion during first 1–2 cycles prior to remission - Do not adjust venetoclax dose for cytopenias during 1st cycle before remission - Patients who experience prolonged cytopenias (e.g., grade 4 lasting >1 week) occurring after remission may require reduction in the *duration* of venetoclax (e.g., reduce by 7 days for the following cycle) and in some cases dose reduction of azacitidine may be necessary - G-CSF may be used following remission to hasten neutrophil recovery for prolonged neutropenia with subsequent cycles - Following remission, delay of next cycle may be required to allow for count recovery
Disease Assessment	- Perform bone marrow aspirate and biopsy ~ day 28 of cycle 1 to assess response. If no CR/CRi recommend to repeat following cycle 2 - Perform bone marrow aspirate and biopsy after remission for suspected relapse or for persistent cytopenias during therapy after remission
